# Biologic therapies for juvenile idiopathic arthritis-associated uveitis

**DOI:** 10.3389/fopht.2022.954901

**Published:** 2022-08-15

**Authors:** Gianluca Dini, Giovanni Battista Dell’Isola, Alfredo Beccasio, Giuseppe Di Cara, Alberto Verrotti, Carlo Cagini

**Affiliations:** ^1^ Department of Pediatrics, University of Perugia, Perugia, Italy; ^2^ Department of Medicine and Surgery, Section of Ophthalmology, University of Perugia, Perugia, Italy

**Keywords:** biologic drugs, juvenile idiopathic arthritis, uveitis, adalimumab, abatacept

## Abstract

Juvenile idiopathic arthritis (JIA) is the most frequent rheumatic disease of childhood and uveitis is its most common extra-articular manifestation. JIA-associated uveitis (JIA-U) is one of the main causes of visual impairment in children and represents a major challenge for pediatrician and ophthalmologist, due to its insidious onset and sight-threatening complications. Topical glucocorticoids are the first line of treatment, followed by conventional disease-modifying anti-rheumatic drugs (DMARDs), usually methotrexate (MTX). In recent years, new biological drugs targeting specific molecules involved in disease pathogenesis, have significantly improved the prognosis of the disease, especially for cases refractory to conventional therapies. In this review we discuss the role of biological agents in JIA-U, focusing on cytokine blockers and cell-targeted therapies aimed to control ocular inflammation.

## Introduction

The term juvenile idiopathic arthritis (JIA) describes a heterogeneous group of chronic arthritis of unknown etiology with onset before the age of 16 (1). The International League of Associations for Rheumatology (ILAR) classified JIA into seven subtypes according to the clinical features of the first six months in the course of the disease (2). Several studies hypothesize an autoimmune pathogenesis of JIA, mediated by B and T lymphocytes activation and the release of pro-inflammatory cytokines such as tumor necrosis factor-alpha (TNFα). The autoimmune reaction seems to arise from the interaction between genetic and environmental factors such as stress, trauma or infections (3). JIA-associated uveitis (JIA-U) is the most common extra-articular manifestation of JIA and the oligoarthritis subtype is characterized by the highest risk of developing uveitis, especially in patients with antinuclear antibodies (ANA) positivity (4). JIA-U is typically non-granulomatous chronic anterior uveitis, using classification criteria proposed by the Standardization of Uveitis Nomenclature (SUN) Working Group (5, 6). The SUN criteria provide a grading system for intraocular inflammation which takes into account the anterior chamber (AC) cells, AC flare, vitreous cells and vitreous haze or debris. This grading system can be used to monitor the disease course and the patient’s response to therapy. JIA-U is associated with a high rate of sight-threatening complications, including posterior synechiae, cataract, glaucoma and band keratopathy (6). JIA-U is often asymptomatic, and its onset can sometimes precede the articular manifestations. Therefore, in order to improve the prognosis of patients and avoid complications, it is essential to carry out an adequate screening program (7). In recent years new biological therapies, targeting immunological pathways involved in disease pathogenesis, have significantly improved the management of JIA-U refractory to conventional therapies. Biological drugs currently used for the treatment of JIA-U include monoclonal antibodies and recombinant proteins that block cytokine activity or modulate lymphocyte functioning (8) (**Table 1**). In this narrative review, we summarize the current management and treatment options of JIA-U, in the context of multidisciplinary care from both rheumatologist and ophthalmologist.

**Table 1:** Biologic drugs used in JIA-U.

Drug	Target	Pediatric indication^1^	Administration and dosage	Side-effects	Reference
**Adalimumab**	TNFα	Non-infectious intermediate, posterior and panuveitis (≥2 yrs) pJIA (≥2 yrs) ERA (≥6 yrs)* PsO (≥4 yrs)* CD (≥6 yrs) UC (≥5 yrs) HS (≥12 yrs)	s.c. (80 mg loading dose, followed by 40 mg on day 8 and every 1-2 weeks thereafter)	Injection site reaction, gastrointestinal disorders, infections, infusion reactions, reactivation of latent tuberculosis	(9)
**Infliximab**	TNFα	JIA-U (off-label) pJIA (off-label) CD (≥6 yrs) UC (≥6 yrs)	IV (loading: 3-5 mg/kg on weeks 0, 2 and 6; maintenance: 3-10 mg/kg every 4-8 weeks depending on response		(10, 11)
**Golimumab**	TNFα	pJIA (≥2 yrs) JPsA (≥2 yrs)	s.c. (50 mg every 4 weeks) or IV (80 mg/m^2^ at weeks 0 and 4, and every 8 weeks thereafter)		(12)
**Etanercept**	TNFα	pJIA (≥2 yrs) JPsA (≥12 yrs) ERA (≥12 yrs)* PsO (≥4 yrs)	Not recommended for the treatment of JIA-U		(13)
**Tocilizumab**	IL-6R	pJIA (≥2 yrs) sJIA (≥2 yrs)	IV (4-12 mg/kg every 2-4 weeks) or s.c. (162 mg every 2 weeks for patients ≥30 kg or every 3 weeks for patients <30 kg)	Liver toxicity (increased plasma ALT), infusion reactions, hypertension, diarrhea	(14, 15)
**Abatacept**	CD80/86	pJIA (≥2 yrs)	IV (10 mg/kg at weeks 0, 2, 4 and continued monthly thereafter)	Headache, bacterial and opportunistic infections, gastrointestinal toxicity	(16)
**Rituximab**	CD20	pJIA (off-label) GPA (≥2 yrs) MPA (≥2 yrs) mature B-cell NHL and mature B-cell AL (≥6 months)	IV (375 mg/m^2^ or 750 mg/m^2^, two doses 2 weeks apart)	Infusion reactions, bacterial infections, neutropenia	(17)
**Baricitinib**	JAK1 and JAK2	The safety and effectiveness in pediatric patients have not been established	Oral (5 mg/day in study)	Infections, headache, cytopenia, hyperlipidemia	(18)
**Tofacitinib**	JAK1 and JAK3	pJIA (≥2 yrs) JPsA (≥2 yrs)	Oral (5 mg twice daily in study)	Upper respiratory tract infections, diarrhea, headache, varicella zoster virus reactivation	(18)

JIA-U, juvenile idiopathic arthritis-associated uveitis; TNFα, tumor necrosis factor-alpha; IL-6R, interleukin-6 receptor; JAK, Janus kinase; pJIA, polyarticular JIA; sJIA, systemic JIA; ERA, enthesitis-related arthritis; JPsA, juvenile psoriatic arthritis; PsO, plaque psoriasis; CD, Crohn’s disease; UC: ulcerative colitis; HS, hidradenitis suppurativa; GPA, Granulomatosis with polyangiitis; MPA, Microscopic polyangiitis; NHL, Non–Hodgkin’s Lymphoma; AL, acute leukemia; IV, intravenous; s.c., subcutaneous; ^1^FDA/EMA-approved drugs; *only approved by EMA, not approved by FDA.

## Materials and methods

The most recent, quality articles were sourced using PubMed, NCBI, Research gate, Medline, EMBASE and Google scholar. Papers were searched using the following terms: “juvenile idiopathic arthritis and uveitis”, “uveitis and biologic drugs”, “uveitis and adalimumab”, “uveitis and abatacept” and “uveitis and JAK inhibitors”. The literature search was primarily focused on randomized controlled trials (RCTs); however, in the absence of such, only the most recently published case reports, case series, clinical trials and open-label studies published to date were included. Only articles in English were reviewed. The abstracts of retrieved references were reviewed and prioritized by relevant content and by the quality of evidence reported. Reference lists of the selected articles were used to search for further relevant papers. Additional information was also obtained from the websites of US and European Union agencies (US Food and Drug Administration and European Medicines Agency).

### Treatment principles

The treatment of JIA-U aims to control ocular inflammation and prevent potentially disabling complications. The target of therapy is to achieve 0 cells in the AC (SUN AC cell grade 0) in both eyes (19). In 2019, the American College of Rheumatology (ACR) developed guidelines for the medical management of JIA-U. According to these guidelines, topical steroids represent the first-line therapy. Also, in patients with acute uveitis already on systemic therapy for JIA it is indicated to start topical corticosteroids as add-on. If local treatment fails to induce remission or patients need topical steroids for 3 months or longer, systemic therapy is recommended (13). Systemic therapies include corticosteroids, conventional Disease Modifying Anti-Rheumatic Drugs (DMARDs) and biological drugs.

### Corticosteroids

Topical glucocorticoids are the initial treatment of both chronic and acute anterior uveitis. Prednisolone acetate 1% is preferred over difluprednate 0.05% for the better corneal penetration and reduced side effects, although long-term comparison data between the two drugs are lacking. The number of drops to be administered should be adapted to the degree of inflammation. The association with a cyclopegic drug such as tropicamide or cyclopentolate 0.5-1% eye drops is recommended to prevent the formation of synechiae (19). In cases of severe JIA-U and in patients without improvement with topical treatment, systemic corticosteroids either orally (prednisolone 1-2 mg/kg/day) or intravenously (methylprednisolone 20-30 mg/kg/day for 1-3 days) may be used to gain rapid control of active uveitis (7). A retrospective study observed that high doses of intravenous methylprednisolone induce rapid improvement in children with non-infectious uveitis (NIU) (20). However, prolonged use of both topical and systemic steroids is burdened with several side effects. Elevation of intraocular pressure, cataract and susceptibility to infections are side effects reported with the use of topical steroids. Moreover, eye drop therapy requires frequent applications and is unable to reach to the posterior segment of the eye (21).

The side effects of long-term systemic steroid use are well known and include but are not limited to weight gain, electrolyte disorders, hypertension, hyperglycemia, adrenal insufficiency, infections and neuropsychiatric effects (22). For this reason, corticosteroids should only be used for short periods of time.

### Conventional disease modifying anti-rheumatic drugs

First-line drug for systemic therapy is methotrexate (MTX) with subcutaneous delivery preferred to oral intake (13). Several retrospective studies have confirmed its effectiveness (23, 24). Almost three quarters of patients treated with MTX respond well to therapy with a steroid-sparing effect after 3-9 months of treatment (25). As alternatives to MTX, other non-biological DMARDs for JIA-U are mycophenolate mofetil (MMF), azathioprine, cyclosporine A (CsA) and leflunomide. However, their use is recommended as second-line and in combination with other immunosuppressant drugs (13). In a retrospective study, treatment with CsA resulted in remission of uveitis in only 24% of patients when used in monotherapy and in 48.6% when combined with other immunosuppressive drugs (26). In another study including forty-one children with JIA-U, azathioprine led to uveitis inactivity in 61.5% of patients when used as systemic monotherapy and in 66.7% when combined with other immunosuppressives (27). MMF has demonstrated effectiveness in childhood uveitis, with a steroid-sparing effect achieved in 88% of patients (28), while leflunomide is associated with a significantly higher flare rate compared to MTX (29).

### Biologic drugs

The term ‘biologics’ refers to complex molecules derived from living organisms or created by recombinant DNA technology. Most biologics used in JIA and associated uveitis were originally developed for the treatment of articular manifestations and their use has subsequently been translated for ocular complications of the disease. They are represented by anti-TNF agents, interleukin inhibitors, T-lymphocyte costimulation modulators, Janus kinase inhibitors and B-lymphocyte inhibitors. Despite the effectiveness of traditional DMARDs, a significant proportion of JIA-U patients are unable to tolerate these agents for long periods of time or only experience a partial benefit from their use. According to the European SHARE initiative, in patients with intolerance or insufficient response to MTX, adding or switching to biological treatment is recommended (30). Also, in case of severe JIA-U or sight-threatening complications, starting immediately an anti-TNF antibody (adalimumab or infliximab) in combination with MTX is recommended over MTX as monotherapy (13). The ACR guidelines suggest that in case of failure of a first anti-TNF agent, switching to another anti-TNF drug is recommended over a biologic in another category. In case of inadequate response to MTX and two TNF inhibitors it is recommended to use abatacept or tocilizumab among the biological drugs, and MMF, leflunomide or CsA among the non-biological DMARDs (13).

### Anti-TNF agents

TNFα is one crucial mediator of inflammation, implicated in many immune-mediated diseases. TNFα participates actively in the pathogenesis of ocular inflammation, with high levels detected in the serum and aqueous humor of patients with uveitis (31, 32). In the past decades, successful biologic agents targeting TNF have been developed for several immune-mediated diseases. Although anti-TNF agents share the same mechanism of action, structural differences in the molecules may contribute to a different efficacy profile (33).

Adalimumab (ADA) is a fully human monoclonal antibody that specifically binds to TNFα. ADA is currently the only FDA-approved biologic for the treatment of NIU in adults and pediatric patients 2 years of age and older (34). ADA is administered by subcutaneous injection. The starting dose for adult patients is 80 mg followed by 40 mg on day 8 and 40 mg every 2 weeks thereafter (35). Two placebo-controlled, double-blind randomized trials, the SYCAMORE trial in the United Kingdom and the ADJUVITE trial in France, have shown the effectiveness of ADA in the treatment of JIA-U. In SYCAMORE trial, a total of 90 patients aged 2-18 years with active JIA-U despite the treatment with MTX, were randomly assigned to receive either ADA (20 or 40 mg every two weeks according to body weight) or placebo and followed for up to 2 years. Early results showed that patients receiving ADA therapy had a much lower treatment failure rate (27%) compared to placebo group (60%) and ADA was overall well tolerated. In the ADA group a significant steroid-sparing effect was also observed (36). In ADJUVITE trial, 31 patients with JIA-associated or idiopathic anterior uveitis refractory to topical steroid therapy and MTX were randomized to receive either ADA subcutaneously (at a dose of 24 mg/m2 in patients aged <13 years, 40 mg in the others) or placebo. After two months of treatment, 9/16 patients in the ADA group achieved a reduction in ocular inflammation, compared to 3/15 in the placebo group (9).

Infliximab (IFX) is a chimeric (mouse/human) monoclonal antibody designed to neutralise TNFα. Currently the only pediatric indication of IFX is the treatment of ulcerative colitis and Crohn’s disease for children older than 6 years (37). In this context, IFX is administered as a slow intravenous infusion over 2-3 hours, starting with a loading dose on weeks 0, 2 and 6, followed by maintenance every 8 weeks (10). In a retrospective study conducted by Miraldi, in a cohort of twenty-seven children with refractory NIU (17 with JIA), long-term treatment with IFX (at least 9 months) led to uveitis control in 89% of patients, with a considerable sparing of concomitant immunosuppressant medications. Fourteen of the twenty-seven patients included in the study failed ADA treatment prior to IFX and after changing to IFX, all but one patient achieved uveitis control (11). In an open-label, multicenter cohort study of childhood NIU, the efficacy and safety of ADA and IFX were compared. Sixteen children (12 with JIA) were recruited in the ADA cohort and seventeen children (10 with JIA) were recruited in the IFX group. At 40 months of follow up, 9 (60%) of 15 children receiving ADA compared to 3 (18.8%) of 16 children receiving IFX were still in remission on therapy (38). Similar results emerged from a study conducted by Cecchin (39), in which 59 JIA-U patients were treated with IFX and 95 with ADA. At the 2-year follow up, ADA showed a better remission rate (60.0%) than IFX (20.3%). Moreover, ADA has the advantage that it can be administered subcutaneously and is therefore generally preferred over IFX which must be administered by intravenous infusion.

Golimumab (GOL) and certolizumab pegol may be effective therapies but evidence is still limited (40). GOL is a fully human monoclonal antibody which is able to bind both soluble and transmembrane TNFα (41). In patients with rheumatoid arthritis (RA), GOL is administered as a 50-mg subcutaneous injection once a month (12). Certolizumab pegol is a pegylated antigen-binding fragment (Fab) of a recombinant humanized anti-TNFα monoclonal antibody. The attachment of a polyethylene glycol (PEG) moiety improves pharmacokinetics and bioavailability of certolizumab (42). Lanz et al. treated with GOL ten patients with JIA-U refractory to ADA. Eight of them had an initial response to GOL which was maintained in five patients at the end of follow-up (43). Palmou-Fontana reported complete remission of uveitis after treatment with GOL in four patients refractory to various biologic agents including ADA, etanercept and IFX, while three other patients did not respond to the drug (44).

Etanercept (ETN) is a fully human dimeric fusion protein approved for use in RA, psoriatic arthritis, plaque psoriasis, ankylosing spondylitis and polyarticular JIA (45). This drug functions as a decoy receptor, preventing TNFα from binding its cell-surface receptors (46). While effective for the treatment of articular symptoms, in a randomized controlled trial involving twelve children with active JIA-U, ETN administered subcutaneously at a dose of 0.4 mg/kg did not provide a significant efficacy above placebo (47). Another study conducted by Tynjälä et al. on forty-five patients with uveitis refractory to combination of DMARDs and steroids showed a greater efficacy of IFX than ETN in controlling ocular inflammation (48). In general, ETN is associated with lower rates of treatment success when compared to ADA and IFX and should be avoided for the treatment of JIA-U (13). A meta-analysis by Li and colleagues (40) compared the efficacy of different TNFα inhibitors in patients with JIA-U. The study evaluated a total of 399 patients, of which 201 were treated with ADA, 139 with IFX, 36 with ETN, 20 with GOL, and 3 with certolizumab pegol. ADA showed significantly higher efficacy than the other TNF inhibitors, with 82% of patients achieving ocular inflammation control versus 56% and 38% for IFX and ETN respectively. The effectiveness of IFX was higher than ETN with no statistical difference (P=0.71). However, in these studies comparing ADA to IFX, the doses of IFX used were generally far lower than typical treatment practice in United States, where IFX is typically used at a minimum of 5 mg/kg every 4 weeks. Although TNF blockers are effective in many cases, patients must be carefully monitored for potential side effects such as a greater risk for infections, anemia and cutaneous reactions. Tuberculosis and viral hepatitis B and C screening procedures are mandatory before starting a TNF blocker (49). Moreover, anti-TNFα monoclonal antibodies such as IFX and ADA may elicit an immune response responsible for the production of antibodies directed against the drug itself. These anti-drug antibodies may reduce the serum concentration of the drug below the threshold of therapeutic efficacy with consequent lack or loss of response (50). In case of lack of efficacy, testing for anti-drug antibodies and measuring the serum concentration of the drug is recommended (30).

### Interleukin inhibitors

Tocilizumab (TCZ) is a recombinant humanized anti-interleukin-6 receptor (IL-6R) monoclonal antibody approved by the FDA for the treatment of RA and for polyarticular and systemic JIA (51, 52). Currently, STOP-Uveitis is the only randomized controlled trial of TCZ in patients with NIU (14). Thirty-seven patients were randomized 1:1 to receive either 4 or 8 mg/kg of intravenous TCZ (IV-TCZ) every 4 weeks for 6 months. In both groups, treatment with TCZ resulted in a significant reduction in vitreous haze and central macular thickness with a corresponding improvement in visual acuity. APTITUDE study was a multicenter phase II clinical trial aiming to explore safety and efficacy of TCZ in children with JIA-U refractory to both MTX and TNF inhibitors (15). Twenty-one patients aged 2-18 years received subcutaneous TCZ at a dosage of 162 mg, administered every 2 weeks in patients weighing more than 30 kg or every 3 weeks in patients weighing less than 30 kg. Although the primary endpoint was not met, one-third of participants responded to TCZ treatment, suggesting TCZ may represent a therapeutic option in some children with uveitis refractory to anti-TNF agents. In a retrospective study, seventeen patients (mean age 15.3 years) with JIA-U refractory to corticosteroids, MTX and other DMARDs including at least one TNFα blocker, were administered IV-TCZ at a dosage of 8 mg/kg every 4 weeks. Ten patients achieved uveitis inactivity after a mean of 5.7 months while a corticosteroids or immunosuppressive treatment sparing was reported in thirteen of the seventeen studied patients. In addition to the beneficial effect on ocular inflammation, efficacy has also been reported on articular manifestations, with active arthritis present in eleven patients at baseline and in six at the last follow-up visit (53).

Di Pasquale et al. described two patients with JIA-U unresponsive to corticosteroids, MTX and TNFα inhibitors, in whom TCZ led to the remission of uveitis after a mean treatment period of 3 weeks (54). Another small case series reported on the use of IV-TCZ in patients with refractory JIA-U. The eight patients in this series were started on IV-TCZ because of inadequate response to at least one conventional immunomodulatory therapy and at least one biologic agent. IV-TCZ was able to induce and maintain remission in five patients. Two patients enrolled in the study had previously received subcutaneous TCZ. One of them achieved remission after IV-TCZ employment, suggesting a greater efficacy of intravenous administration (55).

### T-lymphocyte costimulation modulators

Abatacept (ABA) is a selective T cell costimulation modulator approved for the treatment of both RA and JIA (56). ABA inhibits T cell activation by binding to CD80 and CD86 receptors on antigen-presenting cells (57). ABA is available as an intravenous formulation, administered monthly according to a weight-tiered dosing regimen. In 2008, Angeles-Han first reported the use of ABA for the treatment of refractory JIA-U (58). In 2010, Zulian used intravenous ABA in seven patients with severe, anti-TNF-refractory JIA-U. In all of them, treatment with ABA (10 mg/kg monthly) reduced frequency of uveitis flares and resulted in a significant improvement in the mean AC cell grading (16). Kenawy reported two patients with JIA-U refractory to various antimetabolites and TNF inhibitors, in which ABA infusion induced resolution of the ocular inflammation after 2 months of treatment. Uveitis was inactive in both patients at 12-month follow-up (59). In contrast, in a series of twenty-one patients with severe refractory uveitis, treatment with ABA led to uveitis inactivity in eleven patients, but eight of them later relapsed, while uveitis remained active in the other ten patients (60). In 2011, Elhai described a sustained improvement after ABA treatment in two cases of JIA-U refractory to immunosuppressive drugs and anti-TNFα agents. Moreover, treatment with ABA maintained a good efficacy in controlling the disease even when the intervals between administrations were extended to 6 and 7 weeks apart, respectively (61). In a retrospective study conducted by Birolo (62), ABA showed a comparable efficacy when used as either a first-line biological treatment (n=14) or as rescue therapy after one or more anti-TNF agents (n=17).

### Janus kinase inhibitors

The JAK family is essential for the signal transduction of a wide range of cytokines. Therefore, JAKs-inhibitors appear to have a potential position in the treatment of many autoimmune diseases (63). JAKs form a family consisting of four members: JAK1, JAK2, JAK3 and TYK2, with each having specificity for a different set of cytokine receptors. JAKs inhibitors are not all the same and target different cytokine signalling pathways depending on the specific JAKs targeted. Thus, efficacy and side effects profile are specific to the specific JAK inhibitor. In terms of selectivity, baricitinib has a higher affinity for JAK1 and JAK2 while tofacitinib preferentially inhibits JAK1, JAK3, and to a lesser degree JAK2. Tofacitinib and baricitinib are both approved for the treatment of RA. However, evidence of the efficacy of JAK inhibitors in the treatment of patients with JIA-U is still scarce. Bauermann reported the case of a 22-year-old patient achieving an improvement of uveitis and macular edema after treatment with tofacitinib, while several other DMARD therapies including biologicals, failed to control uveitis activity and edema (64). Miserocchi and colleagues successfully treated with JAK-inhibitors (baricitinib 4 mg/day or tofacitinib 10 mg/day) four patients (aged 18-43 years) with JIA-U refractory to multiple immunosuppressive agents. All patients showed improvement of uveitis and no systemic side effects were reported (18). Currently, an ongoing clinical trial is evaluating the role of baricitinib for ANA positive, early onset, idiopathic or JIA-associated uveitis (NCT04088409) (65).

### B-lymphocyte inhibitors

Rituximab (RTX) is a chimeric mouse-human monoclonal antibody directed against the CD20 molecule, a protein expressed on the surface of mature B cells (66). Immunohistochemical analysis of enucleated eyes and iridectomy specimens of JIA-U patients, revealed the presence of B-cells and fully differentiated plasma cells in the inflammatory ocular cellular infiltrate (67, 68). Moreover, the association of uveitis with ANA-positivity suggests a pathogenetic role of B-cells (69). The administration of intravenous RTX induces a profound depletion of B-cells from the peripheral blood (70). RTX may therefore be helpful for JIA-U that does not respond to corticosteroids, MTX, and anti-TNF agents. In adult patients the rheumatologic protocol includes a single course of two 1000-mg infusions, given 2 weeks apart (71). RTX is not commonly used to treat JIA-U and data present in the literature are mainly derived from small case series. Miserocchi described efficacy of RTX on a cohort of eight patients with JIA-U refractory to conventional immunosuppressants and biologic therapy including TNFα blockers or ABA (17). In a case series of ten patients with JIA-U refractory to other second-line immunosuppressive drugs, a single treatment course of two RTX infusions (375 mg/m2 body surface), two weeks apart, resulted in uveitis remission in seven of them (72). By contrast, in other anecdotal reports RTX has shown a poor efficacy for treating JIA-U (73, 74).

### How long biologic therapy should be continued?

The time point to start tapering and discontinuing biologic agents remains a question. Although consensus recommendations suggest continuing treatment for at least 2 years after uveitis has been controlled (19), there are no well-defined guidelines regarding the exact duration of systemic therapy. In literature there are limited data regarding discontinuation of anti-TNF drugs in patients with JIA-U. In a retrospective cohort study, of eighteen uveitis patients who had discontinued IFX therapy, eleven (61%) relapsed after a median of 603 days and those with JIA-U (n=4) relapsed significantly faster (median time: 76 days) (75). Simonini observed that among children who achieved uveitis inactivity within 6 months, patients treated with anti-TNFα drugs were more likely to maintain remission after therapy discontinuation than those who received MTX (76). Adalimumab in JIA-associated Uveitis Stopping Trial or ADJUST study (NCT03816397) is an on-going trial aiming to provide information regarding discontinuation of ADA in patients with controlled JIA-U. The trial will compare the recurrence of ocular inflammation in JIA-U patients who continue ADA versus those who discontinue ADA and receive placebo over a period of 12 months (77).

## Conclusions

Despite many advances in recent years, treatment of JIA-U remains challenging for clinicians. Since patients are typically asymptomatic, regular ophthalmic screening of children with JIA is highly recommended. Topical glucocorticoids are the initial treatment to achieve control of inflammation, while MTX is the first-line corticosteroid-sparing drug. Biological therapies have significantly improved the prognosis of the disease, especially for children who do not respond or incompletely respond to conventional therapies. In this review we highlighted the advantages and side effects of the biological DMARDs, which are on the market or in the development stage, including blockers of TNF, IL-6R, JAKs, T cells, and B cells. Currently, ADA is the only FDA/EMA-approved biologic for the treatment of NIU. ADA, in addition to having shown greater efficacy than IFX, has the advantage of being administered subcutaneously, while IFX is administered intravenously. However, in studies comparing ADA to IFX, the doses of IFX used were generally far lower than typical treatment practice in United States, where IFX is typically used at a minimum of 5 mg/kg q4 weeks. IFX is considered as second choice and represents a viable alternative for those who had failed treatment with ADA, while ETN should not be considered for the treatment of JIA-U. TCZ and ABA are potential rescue therapies for patients who did not respond to anti-TNF agents. Few uncontrolled studies have highlighted the usefulness of RTX and JAK inhibitors. Future controlled studies are required to confirm the efficacy and safety of these novel therapies. In the management of uveitis, a future goal concern the research of biomarkers in order to be able to predict prognosis and therapeutic response. Regarding the duration of therapy with biologics, this aspect remains unclear and further studies are needed to establish the time point to start tapering or discontinuing the therapy in patients who achieved uveitis inactivity. Long-term safety of biologics must be thoroughly investigated, especially since children with JIA-U require years of treatment.

## Author contributions

GD, GBD and AB put forward the conception of the review and wrote the manuscript. AV and CC participated in the proposal of the concept and revised the manuscript. GD proposed suggestions for revision. All authors approved the submitted version.

## Conflict of interest

The authors declare that the research was conducted in the absence of any commercial or financial relationships that could be construed as a potential conflict of interest.

## Publisher’s note

All claims expressed in this article are solely those of the authors and do not necessarily represent those of their affiliated organizations, or those of the publisher, the editors and the reviewers. Any product that may be evaluated in this article, or claim that may be made by its manufacturer, is not guaranteed or endorsed by the publisher.
